# Is dancing an effective intervention for fat loss? A systematic review and meta-analysis of dance interventions on body composition

**DOI:** 10.1371/journal.pone.0296089

**Published:** 2024-01-17

**Authors:** Yaya Zhang, Zhicheng Guo, Yin Liu, Yongxu Zhou, Longjun Jing

**Affiliations:** 1 School of Physical Education, Hunan University of Science and Technology, Xiangtan, China; 2 Clinical Research Innovation and transformation Center, Zhangjiagang First People’s Hospital, Suzhou, China; Pozan University of Physical Education, POLAND

## Abstract

**Objective:**

The systematic review aimed to review the research on the effects of dance interventions, relative to normal lifestyles, on body composition in people with overweight and obesity.

**Methods:**

7 databases were searched from their inception to 3 July 2023 for studies with dance interventions and normal lifestyles groups. Only studies investigating dance interventions in people with overweight and obesity(body mass index (BMI)>24kg/m2 and percent fat mass (Fat(%)) abnormal(male>20%, female>25%)) were included in the meta-analysis. There were no restrictions on dance forms.

**Results:**

654 studies were identified from the databases, and 10 studies were evaluated to be eligible. The meta-analysis revealed that compared to normal lifestyles dance had meaningful improvements in body mass(BM), BMI, waist circumference(WC), Fat(%), and fat mass(Fat(kg)). No significant differences were found in the waist-to-hip ratio(WHR).

**Conclusions:**

Dance is effective on fat loss in people with overweight and obesity, and has a significant improvement on body composition and morphology. For its high efficiency and greater sense of enjoyment, dance can be a beneficial exercise intervention for fat loss.

## Introduction

With the advancement of socio-economic development, obesity has emerged as a prominent and pressing issue within the realm of global public health [1]. Several studies suggest that obesity is a complex and multifactorial chronic condition intricately associated with factors encompassing the environment [2] (including social and cultural milieu), genetics [3], physiology [4], metabolism [5], and psychological [6] aspects. Furthermore, obesity is significantly correlated with various diseases such as diabetes, gallstones, hypertension, and cardiovascular ailments [7]. The impact of obesity extends across multiple dimensions for individuals afflicted by this condition. Beyond its direct effects, it encompasses a range of potential influences intertwined with daily life. As obesity becomes increasingly severe, the underlying influences also become more pronounced.

To prevent, alleviate, and address the hazards posed by obesity, physical exercise, particularly structured exercise training, remains one of the foremost therapeutic modalities. Regular aerobic exercise performed every week can effectively facilitate weight loss [8], while concurrently enhancing cardiopulmonary fitness and kineticism [9]. However, initiating physical activity is not the primary challenge; the crux lies in sustaining long-term exercise habits [10]. Participants who derive enjoyment from physical activity are more likely to maintain, which indicates the pivotal role of enjoyment as a key factor in sustaining physical exercise [11]. As a form of physical activity that integrates exercise, entertainment, and sociality [12], dance possesses innate advantages in fostering motivation for exercise.

Dance, functioning as an artistic mode of expression, boasts a wide-ranging audience and holds considerable aesthetic value [13]. It can be practiced alone or performed in groups. Different styles of dance vary in their demands for physical movement patterns and degrees of technical proficiency [14]. Moreover, its almost non-existent requirement for specific exercise environments allows dance to cater to the exercise needs of participants with various health conditions, and help them develop term long-term exercise habits.

Growing evidence indicates that the benefits stemming from dance are in both physical and mental dimensions, and these advantages are not limited to specific people. Compared to the non-exercise group, dance can ameliorate VO_2peak_ [15], blood pressure [16], insulin sensitivity [17], physical fitness [18], cognitive disorders [19], and mental health [20]. For physically inactive females, Ljubojevic et al. [21] observed that an 8-week Zumba achieved effective enhancements in body composition and respiratory functionality. Similar improvements were also identified within the diseased population. A pilot study demonstrated that dance can improve body mass index(BMI) and body fat percentage(Fat(%)), while also enhancing their physical activity [22]. People with Parkinson’s disease can achieve physical(balance, functional mobility, and cognition) [23] and mental(self-esteem, quality of life, and motor symptoms) [24] improvements from dancing.

Recent years, various forms of exercise have been substantiated to significantly ameliorate body composition. However, conventional exercise (such as running, cycling, and swimming) is excessively monotonous, posing challenges for adherence. Dance, as a form of exercise engaging multiple joints, not only proves to be efficacious in fat reduction but also boasts amusement value, rendering it more conducive for people to exercise habit formation. Despite clear evidence having substantiated manifold benefits of dance for people. There is no consensus on the specific impact of dance on body composition. The meta-analysis was conducted to 1) explore the effect of dance(including traditional dance and creative dance) on fat loss in people with overweight and obesity; 2) identify people suitable for dance fat loss and the appropriate form and duration of dance for fat loss.

## Materials and methods

The systematic review adhered to the PRISMA-P statement guidelines [25] and was duly registered with the International Prospective Register of Systematic Reviews (PROSPERO) under the registration number CRD42023443866. A PRISMA [26] flowchart was used to depict the details of the study.

### Literature search strategy

A throughout search of the electronic literature was carried out up to 3 July 2023, including Pubmed, Embace, the Cochrane Library, Web of Science, CNKI, Wanfang, and CBM.‘Dance’, ‘Ballet’, ‘body composition’ and so on chosen as the key phrases. The full search strategy was provided in the S1 Data. Searching results were imported into a reference manager(Endnote 20). To make sure comprehensive relevant studies were included in the review, the references list of the eligible studies were also examined. The papers were appraised by 2 researchers independently. By thorough screening, the papers that fit our criteria remained. Any issues in the search process were solved by a third researcher through conversation.

### Inclusion and exclusion criteria

All the researchers were screened following the criteria shown in S1 Table. The following PICOS criteria were used on the screen:

#### Participants

Only subjects with overweight and obesity(BMI>24kg/m2 or Fat(%) abnormal (male>20%, female>25%))were included. People with serious diseases and animal-based subjects were left out. Participants with medical comorbidities were indicated in the result.

#### Intervention

The intervention of the included studies was only dance. No limitation was set on the intensity and form of dance. Participants were undergoing at least 4 weeks of dancing intervention. Any combined intervention(e.g., dancing combined with resistance training) was eliminated during the screening.

#### Comparison

The included studies comprised a control group that undertook a normal lifestyle or normal physical activity. There was no detailed restriction on the control group. Normal physical activity was also appropriate amounts and forms that people can carry out in daily life.

#### Outcomes

The primary data related to fat loss(body mass(BM), BMI, waist circumference(WC), Fat(%), fat mass(Fat(kg)), and waist-to-hip ratio(WHR) were included. The outcomes were all directly reported in the studies, the recalculated values were excluded.

#### Study

Research involving randomized controlled trials(RCT) written in English and Chinese. Books, Observational studies, reviews, and studies without adequate data were left out.

### Data extraction

The data were extracted by 2 researchers independently, and the results were checked by another 2 to ensure the accuracy of the data. The basic characteristics including age, sex ratio, form of dance, intervention characteristics(duration, length, and frequency of dance), and dropouts(both dance and control group) were extracted. The mean ± standard deviation(SD), standard error(SE), or 95% confidence intervals(95%CI) of changes between pre and post-intervention(if not reported, the changes were calculated through the pre and post-intervention data) were extracted. The corresponding author of the included studies was contacted, if the reported outcomes were insufficient or hard to extract. The investigator was requested to provide the data which our analysis required by e-mail. The request lasted for one month, and the study with no response in 1 month was excluded.

### Quality assessment

The appraisal was carried out by 2 researchers. The risk of bias assessment was implemented following a “risk of bias” approach, which is recommended by the Cochrane risk of bias tool [27]. Eligible Studies were examined for seven criteria including random sequence generation, allocation concealment, blinding, incomplete outcome data, selective reporting, and other biases; each evaluation result was rated as “high risk, low risk, and unclear based” on related criteria.

### Statistical analysis

Revman 5.4 and Stata 15 were applied to perform the meta-analysis in this review. The mean±SD of the changes was used to compare the between-group differences. Considering the differences between dance interventions this review included(forms, duration, and so on), the random effect model was adopted for all the outcomes. The mean difference(MD) was used to complete the effect size(ES) if the units of the outcome in the included studies were the international uniform standards. If not, the standardized mean difference(SMD) would be applied. The heterogeneity among studies was quantified using Cochran’s Q test and the inconsistency I2 test. The sensitivity analysis was conducted to explore the heterogeneity. The publication bias was assessed through the funnel plots and Egger’s test.

### Subgroup analysis

To further test the sensitivity, we carried out several subgroup analyses. The analyses were performed to investigate whether the differences between the participants and the dance interventions can influence the final result. Age(≤45 years for the young and >45 years for the middle-aged and older), dance forms(normal dance and creative dance), Comparision(normal lifestyle and exercise), and Duration(<3 months and ≥3 months) were assigned as subgroups.

### Certainty assessment

The GRADEpro GDT [28] was applied to evaluate the certainty of the outcomes. The summary of findings was presented in the result in the form of a table. 2 assessors evaluate independently the risk of bias, inconsistency, indirectness, imprecision, and other considerations of all the included RCTs following the guideline [29]. Any arguments were solved by 1 researcher through conversation.

## Result

### Included studies

**Fig 1:**
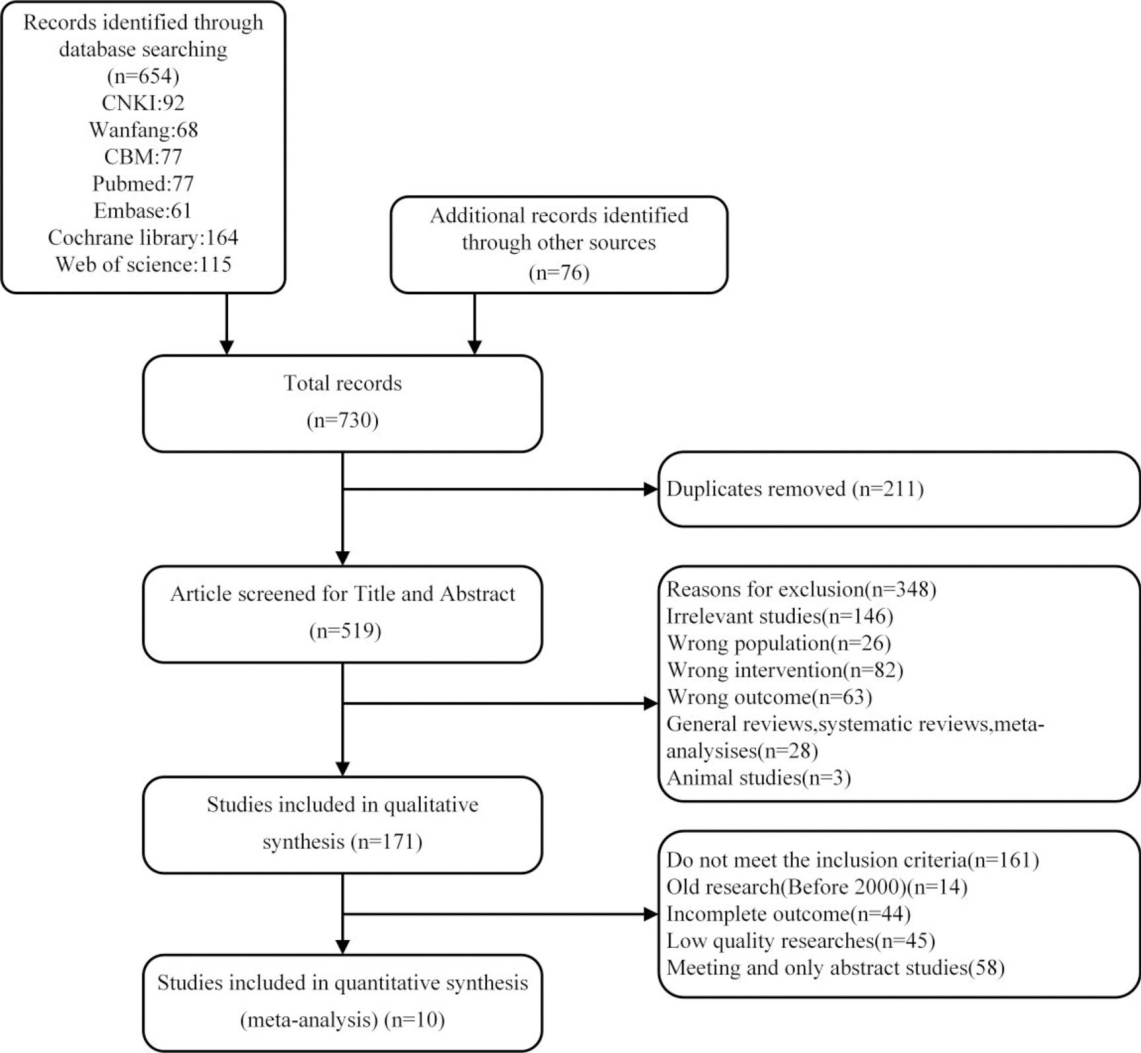
Literature search and study selection process.

After comprehensive searching, 654 studies were retrieved from 7 databases and 76 studies from references and other sources. A total of 730 researches were imported in Endnote 20. 211 duplicates were removed, and 519 studies were remained. After evaluating the title and abstract, 348 studies were excluded. After full-text screening, 161 researchers did not meet the inclusion criteria. At last 10 studies passed the evaluation and were included for further analysis. (The complete screening is shown in Fig 1.)

### Participant and intervention characteristics

646 participants with overweight and obesity in 10 studies [30–39] were included in the analysis. As summarized in Table 1, 321 participants were allocated to the dance program, and 331 were to the control group. The age of the eligible participants was 48.22±5.01 years. The sex ratio of the included studies was nearly 1:5. Among the included studies, only three applied traditional dance, the forms of the other seven were more innovative and creative. Most frequency of interventions were 3 times/week. The duration of six interventions exceeded ≥ 60 mins, and the shortest intervention was at 40 mins. Most control groups were asked to maintain their normal lifestyles. After reading the full text, the intensity of the control group exercise in the included studies [34, 35] was within the World Health Organization(WHO) recommended weekly exercise range(a minimum of 150–300 mins of moderate-intensity exercise [40]). So we did not exclude the studies with exercise as the control group. Half studies had dropouts, while no studies had a> 85% attendance rate.

**Table 1:** Characteristics of included studies.

Study	Sample size (Intervention/Control)	Sex (male/female)	Age(years)	Forms of dance	Control group	Frequency	Duration	Intervention length	Total Dropouts (dance/control;)
Arslan 2011	29/20	0/49	41.55 ± 6.72	Step-aerobic dance	Maintain normal lifestyles	3 times/week	40–50 mins	8 weeks	0/0
Chen 2016	90/90	0/180	21.43±1.19	Dance Cheerleading	Normal physical education	3 times/week	90 mins	14 weeks	0/0
Cruz-Ferreira 2015	32/25	0/57	71.95±4.2	Creative dance	Maintain normal lifestyles	3 times/week	50 mins	12weeks/24 weeks	0/0
Domene 2016	11/11	0/20	34±12	Zumba	Maintain normal lifestyles	3 times/week	60 mins	8 weeks	1/1
Mangeri 2014	42/58	52/48	58.95±8.65	Bhangra dance	Self-selected physical activity	2 times/week	60 mins	12weeks/24 weeks	1/6
Miyazaki 2022	30/29/29	62/26	67.81±5.69	Traditional dance	Walking/Maintain normal lfiestyles	3 times/week	45 mins	4 weeks	0/2/0
Staiano 2017	22/19	0/41	16±1.4	Dance video games	Maintain normal lifestyles	3 times/week	60 mins	12 weeks	2/1
Wang 2020	15/15/15	0/45	58.07±3.18	Square dance	Maintain normal lifestyles	5 times/week	60 mins	6 weeks	0/0/0
Wang 2023	15/15	0/30	70±2.34	Simplified dance	Maintain normal lifestyles	3 times/week	45 mins	12 weeks	1/3
Zhang 2008	20/20	0/40	42.4±5.32	Aerobic fitness dance	Maintain normal lifestyles	3 times/week	90 mins	1 year	0/0

### Outcome assessment

The BM and height of participants were measured directly through a digital scale and a stadiometer. The BMI was calculated as weight divided by height squared (kg/m2). The WC was measured by a tape meter at the natural waist. The hip circumference was evaluated at the level of the maximum extension of the thigh. The WHR was calculated as WC divided by the hip circumference. Of the included studies, 3 studies applied bioelectrical impedance analysis(BIA) to measure the Fat(kg) and Fat(%), 1 study used dual-energy X-ray absorptiometry (DXA), and 1 study did not report the measurement method.1 study did not report Fat(kg), and only report Fat(%) by measuring the skinfold thickness.

### Quality assessment

As S1 and S2 Figs presented, 10 studies were assessed. 6 studies reported allocation plan(3 concealed allocation, 3 did not), and 4 studies did not report. All the participants in the dance group knew that they were performing a wonderful aerobic exercise, the blinding of participants was impossible. Half studies did not illustrate whether the assessment was blinded. In another half, only 1 study did not blind the outcome evaluators. The dropouts of 3 studies might influence the completeness of the outcomes. No studies had selectively reported, and the designs of the 3 studies were special which might cause unclear bias.

### Meta-analysis

**Fig 2:**
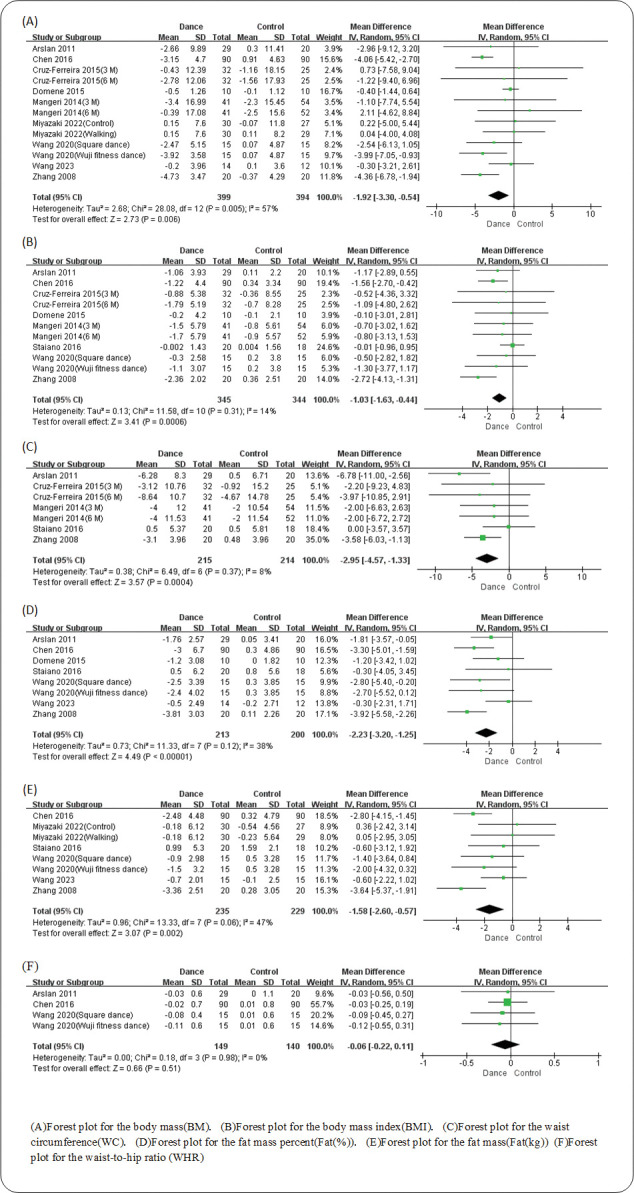
Forest plot for dance vs control group.

All 10 studies examined the effects of dance programs versus the control group on body composition. 9 studies reported BM, 8 reported BMI, 5 reported WC, 7 reported Fat(%), 6 reported Fat(kg), and 3 reported WHR. Based on the 9 eligible studies examing BM, the result of the meta-analysis identified a significant pooled MD estimate(MD = -1.92kg; 95% CI -3.3 to -0.54; p = 0.006), which suggested that dance programs had a better influence on BM improvement compared to the control group. Evidence that dance achieves great benefit on fat loss were also found in other body composition variables. Significant differences were also found in BMI(MD = -1.03kg/m2; 95% CI -1.63 to -0.44; p = 0.0006), WC(MD = -2.95cm; 95% CI -4.57 to -1.33; p = 0.0004), Fat(%)(MD = -2.23%; 95% CI -3.2 to -1.25; p<0.00001), Fat(kg)(MD = -1.58kg; 95% CI -2.6 to -0.57; p = 0.002), which indicated that dance can achieve comprehensive improvement on body composition. However, in WHR there were no statistical differences(MD = -0.06; 95% CI -0.22 to 0.11; p = 0.51) between the dance group and the control group (Fig 2).

### Sensitivity analysis and publication bias

As the Fig 2 shown, the heterogeneities of BM(I^2^ = 57%), Fat(%)(I^2^ = 38%), and Fat(kg)(I^2^ = 47%) were abnormal. The heterogeneities of BMI(I^2^ = 14%), WC(I^2^ = 8%), and WHR(I^2^ = 0%) were low. After eliminating the included studies respectively, we found that the heterogeneity was from 2 studies [31, 39] (both due to unique dance design). No significant asymmetry was found in the funnel plots (S3 Fig), which indicated no significant publication bias.

### Subgroup analysis

The subgroup analysis was performed only when ≥ 2 studies were included in the subgroup. As S2 Table showed, statistical differences(p < 0.05) were found between the dance group and the control group in most ≤45 years, creative dance, normal lifestyle, and ≥ 3 months subgroups on body composition. A significant effect was found on BMI and WC respectively in the exercise subgroup and the < 3 months subgroup. On Fat(%), the results of 7 subgroup analyses(except normal dance) revealed statistical differences, which identified that no matter what participants and intervention duration dance had a meaningful effect on fat loss.

### GRADE assessment

As S3 Table summarised, the GREADpro system was used to evaluate the certainty of evidence of outcomes BM, BMI, WC, Fat(%), Fat(kg), and WHR. The quality of the overall outcomes was low. The different forms of dance were the leading cause of most downgrades. In terms of the quality of the evidence, except the BM, BMI, and WC were moderate, Fat(%), Fat(kg), and WHR were low or very low, which was probably due to the outcomes bias of 1 study [30].

## Discussion

This systematic review was conducted to determine the effect of dance on fat loss. To our knowledge, this is the first systematic review focusing on the effectiveness of dance intervention on body composition. In this review, 10 studies involving 646 participants were included in the meta-analysis. The results indicated that when compared to the normal lifestyle, dance exhibits a significant effect on the improvement of body composition among people with overweight and obesity. From the meta-analysis, dance demonstrated the capacity to effectively reduce BM, BMI, WC), Fat (%), and Fat(kg), which was in agreement with previous researches [41–43]. These effects exhibited statistical differences in comparison to the control group(p < 0.05). However, to WHR there was no statistical differences between the dance group and the control group(p > 0.05). This could potentially be attributed to the comprehensive fat reduction effects induced by dance, which is distributed throughout the entire body rather than being concentrated in a specific anatomical region. Subgroup analysis revealed that dance is more suitable for improving body composition and physique among younger individuals (<45 years). In contrast, significant differences were observed only in the FAT (%) subgroup in the middle-aged and elderly(>45 years). Even though no statistical differences were found, dance still caused reductions in various indicators compared to the control group. From the findings within the dance forms subgroups, we concluded that creative dance yields more pronounced body composition improvement. Conversely, the improvements generated by traditional dance appear to be less prominent. This could be due to the ongoing evolution of dance, marked by advancements in both intensity and form [44]. While it should be noted that in this review the control group of 3 included studies [31, 34, 35] were exercise interventions, the intensity of these interventions remained within the range recommended by the WHO. The outcomes of subgroup analysis also indicate that these differences had a limited impact on the overall results of the meta-analysis. Regarding the intervention duration, the findings of this review align closely with previous researches [45–47]. As a form of aerobic exercise, dance necessitates an intervention duration of at least 3 months to yield substantial effects on body composition. The overall dropout of the dance group was low. On the whole, Dance can be effectively advocated as a viable fat loss program for people with overweight and obesity, owing to the inherent enjoyability of dance which makes participants more likely to sustain.

BM and BMI, as two readily available body composition indicators in daily life, have found extensive application in various fat loss regimens. This review demonstrated that dance can lead to significant improvements in body mass and BMI. Such improvements were also been reported in children with overweight and obesity [48] and patients with Parkinson’s disease [49]. However, both BM and BMI had heterogeneity(BM demonstrating notably higher heterogeneity I^2^ = 57%). Heterogeneity was also present within the subgroups that show statistical significance in the subgroup analysis. After excluding studies with high heterogeneity, the results of the meta-analysis remained unchanged. In terms of the included studies, the mechanisms through which dance reduces BM and BMI are aligned with those of aerobic exercise [50]. Due to the various forms of dance and the heterogeneity, the certainty of the evidence of BM and BMI were categorized as moderate. Taken together, we considered dance to be a better form of aerobic exercise for people with overweight to reduce BM and BMI.

Obesity was identified as a prominent risk factor for cardiovascular disease (CVD), and Fat(%)demonstrated efficacy in the predictive prognosis of CVD [51]. Furthermore, the Fat(%) was correlated with the survival rate of patients with CVD [52]. The reduction of Fat(%) can engender a heightened sense of enjoyment and enhance adherence to exercise. The meta-analysis indicated that compared to the control group, dance significantly reduced the Fat(%), and this improvement held notable clinical significance (MD = -2.23%). From the results of subgroup analysis, we observed that the improvement in Fat(%) through dance was scarcely influenced by age, form of dance, and intervention duration. Except the exercise subgroup where assessment of dance effects was inconclusive due to limited inclusion (n = 1), all other subgroups exhibited clinically meaningful reductions in body Fat(%). Similar fat reduction was also observed in aerobic exercise [53], resistance training [54], and high-intensity interval training (HIIT) [55]. However, dance exhibited a distinct adherence advantage in terms of reducing Fat(%). This can be attributed to the fact that dance is characterized by being a full-body exercise. Purely localized body exercises tend to have lower overall energy expenditure and are more prone to fatigue, making them hard to sustain. While the heterogeneity of Fat(%) was within an acceptable range (I^2^ = 38%), the GRADE for this outcome was assessed as very low. Therefore, this result requires careful consideration when applied to practice.

In contrast to dietary interventions, the reduction in Fat(kg) resulting from exercise does not lead to significant loss in fat-free mass(FFM) [56]. The reduction in Fat(kg) induced by dance was directly translated into an improvement in Fat(%). Our results demonstrated that similar to Fat(%), dance also led to a significant reduction in Fat(kg). However, in subgroup analysis, statistical differences were observed only in subgroups ≤45 years, creative dance, normal life, and ≥ 3 months. This suggested that the improvements brought about by dance are comprehensive in terms of body composition. Unlike resistance training, even if not statistically significant, aerobic exercise, including dance, resulted in a certain FFM loss [53, 57]. However, compared to the normal lifestyle, engaging in dance can reduce Fat(kg) while concurrently maintaining a lesser loss of FFM [58]. Given that the heterogeneity of Fat(kg) is approaching moderate(I^2^ = 47%). Even after excluding a study [39] with high heterogeneity, the meta-analysis results remained unchanged. The certainty assessment of Fat(kg) was low. However, we still suggested that dance constitutes a highly effective exercise intervention for reducing Fat(kg) among people with overweight and obesity.

WC serves as a critical indicator for assessing abdominal obesity [59], proving to be an effective method for predicting both obesity and cardiovascular disease risk [60]. The WHR is utilized to assess central fat distribution and stands as the primary metric pursued by dancers for body morphology improvement [61]. In this meta-analysis, both WC and WHR were exhibited to be low heterogeneity (WC: I^2^ = 8%, WHR: I^2^ = 0). There were significant differences between the dance group and the control group in WC. Such differences were not found in WHR. Based on this, we deduced that dance has the potential to concurrently improve WHR in people with overweight and obesity, resulting in a slight reduction in waist-to-hip ratio. Similar improvements were reported in aerobic exercise [62] and HIIT [63]. A recent study that explored the impact of the forms of exercise on obesity indices revealed that dance exhibited a superior efficacy in ameliorating WC and WHR [64]. Given that within the WC subgroup, the studies encompassed in creative dance and normal lifestyle groups were identical, and the < 3 months group included only one study. We only inferred that dance was more suitable for improving WC in the <45 years age people. The subgroup analysis for WHR lacked significant analytical value due to the scarcity of included studies. The evidence grade of WC was moderate, and the WHR was very low. Hence, we speculated that the mechanisms underlying the improvement in WC and WHR through dance were akin to those of aerobic exercises. Further research is needed to validate the improvements in WC and WHR brought about by the holistic bodily movements characteristic of dance.

To elucidate the fat loss effects of dance, we conducted a thorough analysis of relevant research studies. The results indicated that compared to the control group, dance significantly enhanced the body composition of the participants, which was of important clinical significance. However, there were several limitations to be acknowledged in this systematic review. Many researches indicated the presence of gender differences in the effects of exercise on health [65–67]. The majority of participants in dance are women, and the studies included in this meta-analysis also predominantly focus on female subjects(Only 2 studies had male participants included.). This raised questions about the applicability of the findings from this study to male fat loss. The diverse forms of dance are characterized by engaging various body segments in coordinated movement. The diversity of dance forms arise from the characteristic of involving various body segments in coordinated movement. Such diversity could potentially impact the results of the meta-analysis. Our study included at least 7 distinct forms of dance. The heterogeneity of dance forms served as a primary source of bias in this study, influencing the certainty assessment of the outcomes. Conducting subgroup analysis based solely on traditional and creative forms was insufficient to elucidate the variations in the effectiveness of different dance forms. Further research is required to ascertain the differences in the effects on body composition among different dance forms. Finally, although dance can provide participants with a heightened sense of enjoyment. Constrained by the included studies, our study did not analyze compliance with dance. Only the low total dropouts were not enough to substantiate the high adherence to dance. Future research needs to ascertain whether different forms of dance yield similar benefits on body composition. Further comparative analysis between dance and other exercises regarding compliance is warranted, aiming to delve into the origins of enjoyment induced by dance. More studies are needed to explore gender differences in dance, along with clarifying the common advantages of dance.

## Conclusion

Dance can effectively ameliorate body composition among people with overweight and obesity. Simultaneously with fat loss, dance preserves and enhances the body morphology of the participants. Moreover, dance is particularly well-suited for the young population (<45 years) as a substitute for traditional exercise protocols in terms of fat loss. Duration lasting for more than 3 months, along with creative dance forms, is more conducive to achieving clinical objectives related to improvements in body composition. Studies with larger sample sizes, longer interventions, and more comprehensive need to be conducted to provide more compelling evidence to elucidate the high adherence and enjoyment of dance in fat loss.

## Supporting information

S1 Checklist PRISMA 2020 checklist. (DOCX)

S2 Checklist Human participants research checklist. (DOCX)

S1 Fig Summary of risk bias: Review authors’ judgment of risk bias for each item. (DOC)

S2 Fig Review judgment of risk bias for each item. (DOCX)

S3 Fig Funnel plot of dance vs control group. (DOC)

S1 Table Criteria for inclusion and exclusion. (XLS)

S2 Table Summary of dance vs control group subgroup analysis. (XLS)

S3 Table Certainty-of-evidence ratings of studies camparing dance with normal lifestyle. (DOCX)

S1 Data (ZIP)
