# Supplementary material for: Is dancing an effective intervention for fat loss? A systematic review and meta-analysis of dance interventions on body composition
Source: PLoS One. 2024 Jan 17;19(1):e0296089. doi: 10.1371/journal.pone.0296089 (PMC10793915; doi:10.1371/journal.pone.0296089)
Supplement: S1 Fig — (DOC) [file pone.0296089.s003.doc]

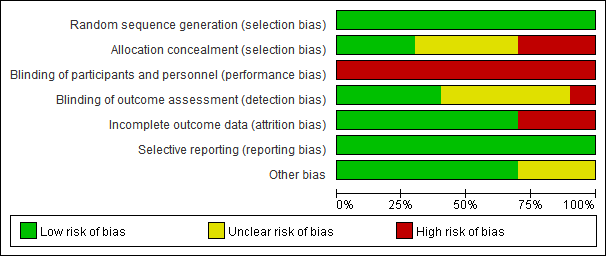


Fig S1 Summary of risk bias: review authors’ judgment of risk bias for each item.

Risk of bias levels: low (green), unclear (yellow), and high (red).
