# Supplementary material for: Is dancing an effective intervention for fat loss? A systematic review and meta-analysis of dance interventions on body composition
Source: PLoS One. 2024 Jan 17;19(1):e0296089. doi: 10.1371/journal.pone.0296089 (PMC10793915; doi:10.1371/journal.pone.0296089)
Supplement: S2 Fig — (DOCX) [file pone.0296089.s004.docx]

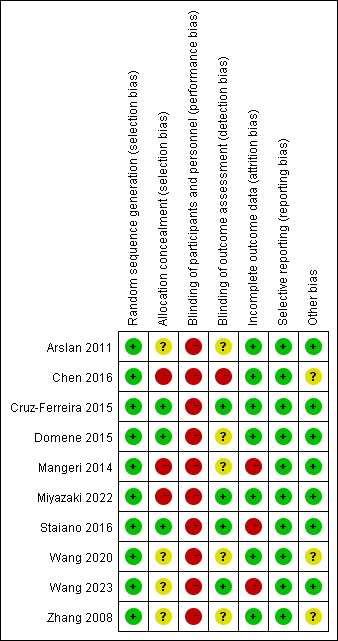


Fig S2 Review judgment of risk bias for each item.

percentages across all included studies. Risk of bias levels: low (green or “+”), unclear (yellow or “?”), and high (red or “−”).
