# Supplementary material for: Is dancing an effective intervention for fat loss? A systematic review and meta-analysis of dance interventions on body composition
Source: PLoS One. 2024 Jan 17;19(1):e0296089. doi: 10.1371/journal.pone.0296089 (PMC10793915; doi:10.1371/journal.pone.0296089)
Supplement: S3 Fig — (DOC) [file pone.0296089.s005.doc]

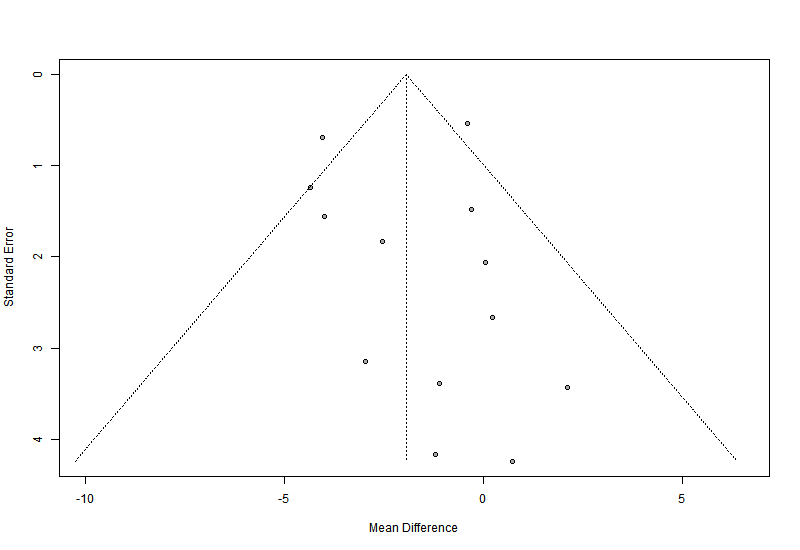


A


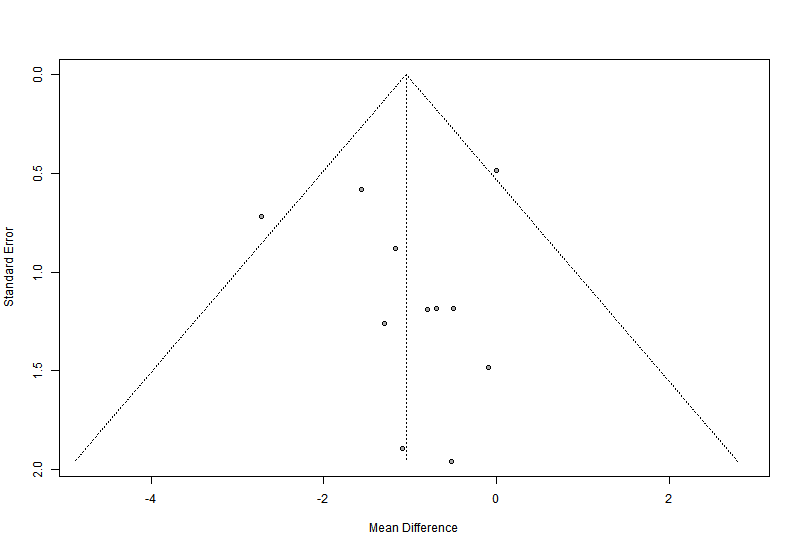


**B**


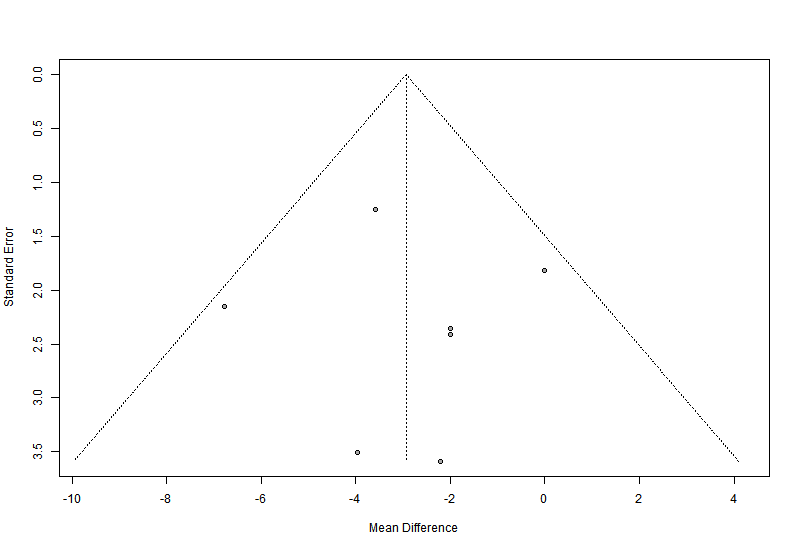


**C**


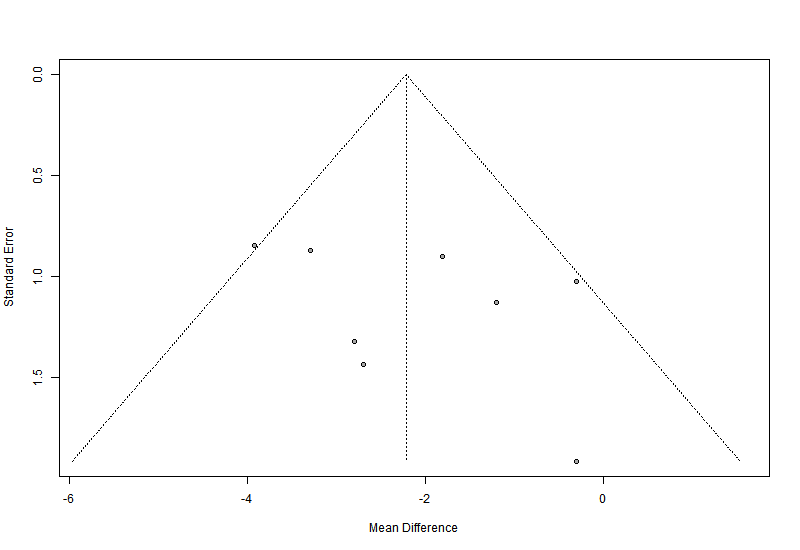


**D**


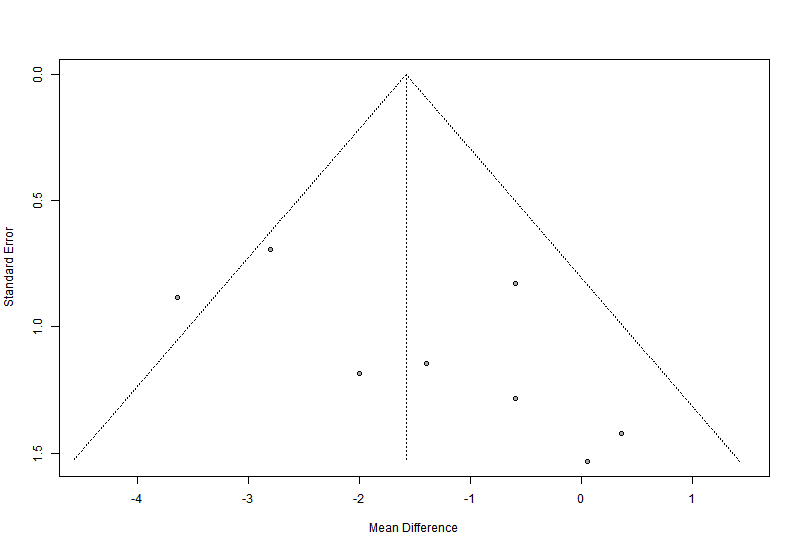


**E**


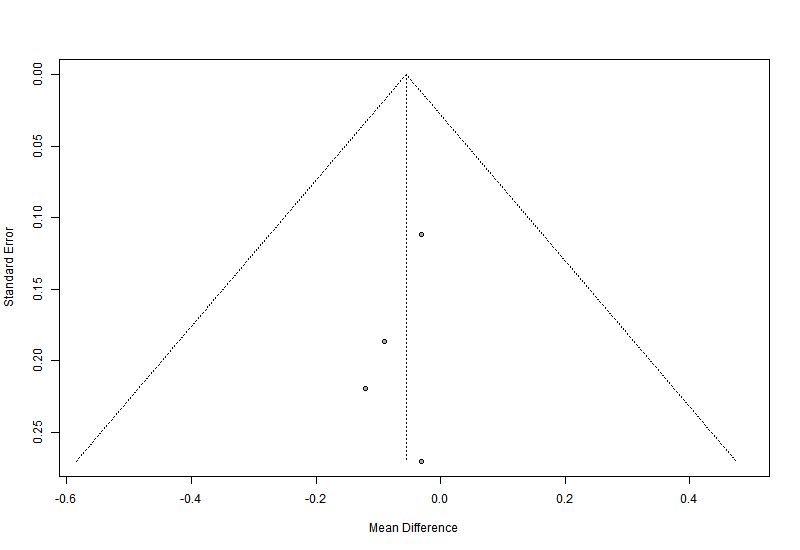


**F**

Fig S3 Funnel plot of dance vs control group.

(A)Funnel plot for BM. (B) Funnel plot for BMI. (C) Funnel plot for WC. (D) Funnel plot for FAT(%). (E) Funnel plot for FAT(kg). (F) Funnel plot for (WHR)

.
