# Supplementary material for: Is dancing an effective intervention for fat loss? A systematic review and meta-analysis of dance interventions on body composition
Source: PLoS One. 2024 Jan 17;19(1):e0296089. doi: 10.1371/journal.pone.0296089 (PMC10793915; doi:10.1371/journal.pone.0296089)
Supplement: S3 Table — (DOCX) [file pone.0296089.s008.docx]

| **Table S3 Certainty-of-evidence ratings of studies camparing dance with normal lifestyle** | | | | | |
| --- | --- | --- | --- | --- | --- |
| **Patient or population:** People with overweigth or obesity(BMI>24kg/m2 or Fat% abnormal(male>20%, female>25%))  **Setting:** Dance compared to the normal lifestyles  **Intervention:** Different forms of dance  **Comparison:** the control group | | | | | |
| **Outcomes** | **№ of participants (studies) Follow-up** | **Certainty of the evidence (GRADE)** | **Relative effect (95% CI)** | **Anticipated absolute effects** | |
|  |  |  |  | **Risk with placebo** | **Risk difference with body composition** |
| BM | 793 (13 RCTs) | ⨁⨁⨁◯ Moderate^a,b,c,d^ | - | The mean BM was **0** | MD **1.92 lower** (3.3 lower to 0.54 lower) |
| BMI | 689 (11 RCTs) | ⨁⨁⨁◯ Moderate^a,b,c,d^ | - | The mean BMI was **0** | MD **1.03 lower** (1.63 lower to 0.44 lower) |
| WC | 429 (7 RCTs) | ⨁⨁⨁◯ Moderate^a,b,c,d^ | - | The mean WC was **0** | MD **2.95 lower** (4.57 lower to 1.33 lower) |
| Fat(%) | 413 (8 RCTs) | ⨁◯◯◯ Very low^a,b,c,d,e^ | - | The mean fat(%) was **0** | MD **2.23 lower** (3.2 lower to 1.25 lower) |
| Fat(kg) | 464 (8 RCTs) | ⨁⨁◯◯ Low^a,b,c,d^ | - | The mean fat(kg) was **0** | MD **1.58 lower** (2.6 lower to 0.57 lower) |
| WHR | 289 (4 RCTs) | ⨁◯◯◯ Very low^b,f,g,h^ | - | The mean WHR was **0** | MD **0.06 lower** (0.22 lower to 0.11 higher) |
| ***The risk in the intervention group** (and its 95% confidence interval) is based on the assumed risk in the comparison group and the **relative effect** of the intervention (and its 95% CI). **CI:** confidence interval; **MD:** mean difference | | | | | |
| **GRADE Working Group grades of evidence** **High certainty:** we are very confident that the true effect lies close to that of the estimate of the effect. **Moderate certainty:** we are moderately confident in the effect estimate: the true effect is likely to be close to the estimate of the effect, but there is a possibility that it is substantially different. **Low certainty:** our confidence in the effect estimate is limited: the true effect may be substantially different from the estimate of the effect. **Very low certainty:** we have very little confidence in the effect estimate: the true effect is likely to be substantially different from the estimate of effect. | | | | | |

#### Explanations：

a. Included study with unclear allocation concealment, decision: downgrade 1 level for risk of bias.

b. Different forms of dacne, decision: downgrade 1 level for inconsistency.

c. Included study with the exercise control group, downgraded 1 level for inconsistency.

d. Included study with drop out, decision: downgrade 1 level for outcomes reporting bias.

e. Included study with unclear Fat(%) measurement method, decision: downgrade 1 level for outcomes bias.

f. Included studies were small, do not meet optimal information size, decision: downgrade 2 level for imprecision.

g. Number of participants < 400., decision: downgrade 1 level for imprecision.

h. The confidence interval reached the null effect, decision: downgrade 1 level for imprecision

#### Abbreviations:CI, Confidence interval; SD, Standard deviations; BM, body mass; BMI, body mass index; Fat(%), percent fat mass; Fat(kg), fat mass; WC, waist circumference; WHR, waist-to-hip ratio.
